# A prevention program for binge drinking among students based on mindfulness and implementation intention (ALCOMEDIIT): a randomized controlled trial

**DOI:** 10.1186/s13063-023-07887-9

**Published:** 2024-01-02

**Authors:** Jessica Mange, Nicolas Mauny, Charlotte Montcharmont, Eve Legrand, Maud Lemercier-Dugarin, Arnaud Mortier, Martin Duvivier, Johnny Leveneur, Cédric Lacherez, Nicolas Cabé, Anne-Pascale Le Berre

**Affiliations:** 1 Laboratoire de Psychologie Caen Normandie (LPCN, UR 7452), University of Caen Normandy, Esplanade de la Paix, 14032 Caen, cedex 5 France; 2 Laboratoire Parisien de Psychologie Sociale (LAPPS), University Paris Nanterre, Nanterre, France; 3 University of Caen Normandy, CNRS, LMNO UMR 6139, F-14000 Caen, France; 4 Information System and Communication Department, University of Caen Normandy, Caen, France; 5 Normandie Univ, UNICAEN, PSL Université, EPHE, NSERM, U1077, CHU de Caen, GIP Cyceron, NIMH, 14000 Caen, France; 6 Department of Addictology, Public Institution of Mental Health (EPSM) of Finistère Sud, Quimper, France

**Keywords:** Prevention, Alcohol, Binge drinking, Motivational interviewing, Mindfulness meditation, Implementation intentions, Brief intervention, Impulsivity, Students

## Abstract

**Background:**

The emergence of new problematic alcohol consumption practices among young people requires new dynamics in prevention strategies. In this context, the ADUC project (Alcohol and Drugs at the University of Caen) aims to develop a better understanding of alcohol consumption, and in particular the practice of binge drinking (BD) in students, in order to develop relevant and adapted prevention tools. The ALCOMEDIIT study (Rin Normandie and IRESP funding; Agreement 20II31-00 - ADUC part 3) is a randomized controlled trial that focuses on the specific determinant of impulsivity. The main objective of this experiment is to assess a program for the prevention of BD practices based on motivational interviewing (MI) associated with implementation intention (II) and mindfulness meditation (MBM) in a student environment.

**Methods:**

This study will include 170 healthy subjects who will be students at the university, alcohol users, with a BD score > 1 in the month preceding the inclusion but not presenting any specific disorder. The trial will be proposed by e-mail and students who meet the inclusion criteria will join either a control group which will benefit from a MI or an experimental group which will additionally benefit from an initiation to MBM with II (initial visit T0). In order to measure the effectiveness of the prevention program in terms of BD decrease, a follow-up at 1 month (T1) as well as a follow-up at 6 months (T6; exploratory) will be proposed to all participants. The total duration of this research protocol is 21 months.

**Discussion:**

The purpose of this study is to evaluate the interest of associating mindfulness meditation practices and implementation of self-regulation strategies to optimize their use, with a motivational interview in an innovative prevention program aiming at reducing alcohol use and BD practice in the student population.

**Trial registration:**

ClinicalTrials.gov Identifier: NCT05565989, September 30, 2022.

https://clinicaltrials.gov/study/NCT05565989

Protocol version 2.0 (September 2022)

No. ID-RCB : 2022-A00983-40

**Supplementary Information:**

The online version contains supplementary material available at 10.1186/s13063-023-07887-9.

## Background

Alcohol is the most consumed psychoactive substance by French adolescents [1]. Although this consumption tended to decrease over the last decade (ibid.), the emergence of new problematic alcohol consumption practices requires new dynamics in prevention strategies. In this perspective, the phenomenon of binge drinking (BD) has mobilized researchers since the 2000s. Binge drinking refers to the consumption of a large quantity of alcohol over a short period of time (6 drinks for women and 7 drinks for men in 2 hours in France). In Europe, the prevalence of BD is about 28% among students (European Union: Directorate General for Communication, 2010). The developing brain is particularly vulnerable to the effects of alcohol [2]. Indeed, if the recreational aspect of the BD may have been an illusion at first, its major consequences in personal, academic, or social terms are now relatively documented [3] as well as the brain damage and cognitive disorders associated with this practice [4–7]. Consequently, alcohol consumption among young people and binge drinking in particular are now a public health issue.

### Prevention, motivational interviewing, and impulsivity

Current prevention techniques are generally classified as environmental (e.g., maintaining a minimum legal drinking age) or individual-focused (e.g., College Alcohol Intervention Matrix, National Institute of Alcohol Abuse & Alcoholism, USA). Among the prevention techniques focused on the individual, reference is often made to methods based on motivational interviewing (MI) [8] aimed at exploring and resolving the ambivalence a person may express about his or her alcohol use in order to initiate a process of behavior change. It is interesting to note that the MI can be run as a single session lasting from 15 to 45 min [9] and that it is optimized in a face-to-face situation. This MI technique, considered by some as one of the most effective prevention techniques with a particularly optimal investment/efficacy ratio [10], is nevertheless discussed [11]. Among the explanations of the heterogeneous results usually observed, two complementary explanations can be proposed. On the one hand, studies testing the effectiveness of MI target beneficiaries with heterogeneous psychological profiles (i.e., with distinct psychological determinants specific to their consumption) in an undifferentiated way. However, various determinants have been highlighted with regard to the problem of the practice of BD [12], and not all of them are necessarily addressed or considered in a MI approach. On the other hand, the MI acts on a first necessary step for change, meaning the awareness of a risky situation, but not on the strategies allowing the implementation of the desired change. Thus, taking these two elements into consideration, we propose to combine the MI with a complementary module specifically targeting an important determinant in the context of alcohol use, namely impulsivity. The critical role of impulsivity has been identified in BD [12] and substance use in general [13]. Indeed, impulsivity, defined as a tendency for individuals to act prematurely without fully considering the consequences of their actions, is considered as a major factor associated with alcohol consumption and BD. As impulsivity cannot be considered as a unitary construct [14], its sub-dimensions have been dissociated to disentangle their respective roles [15]. Today, five facets are considered: negative vs. positive urgency (i.e., the tendency to act impetuously to regulate a negative vs. positive emotion), lack of premeditation (i.e., the tendency to favor immediate reward options without considering the potential consequences of the action), lack of perseverance (i.e., less focusing on boring or difficult tasks and distraction, and lack of interest in the future), and sensation seeking (i.e., the tendency to seek a new or exciting experience).

To target the drinking processes specifically related to impulsivity, Larsen and Hollands’ (2021) theoretical model [16] proposes different prevention techniques to regulate the automatic processes underlying maladaptive behavior that can be harmful to health. The ALCOMEDIIT module is based on two antecedent-focused strategies that aim to avoid exposure to or activation of automatic (i.e., impulsive) responses that lead to alcohol consumption.

### Mindfulness intervention

First, the ALCOMEDIIT module will offer an initiation to mindfulness meditation (MM), a so-called indirect strategy aimed at modifying unwanted automatic responses by strengthening self-control skills, especially by automating self-control processes as well as by facilitating a more efficient executive functioning ensuring cognitive and behavioral control mechanisms. Indeed, a possible tool to fight against impulsivity towards the desire to consume alcohol is MM [17]. MM is described as “the awareness that emerges from purposefully paying attention, in the present moment and without judgment, to the unfolding of experience from moment to moment” [18]. MM-based interventions have already proven to be effective in reducing a variety of substance uses, particularly in an adult population [19]. More specifically, the Mindfulness Based Relapse Prevention (MBRP) program has demonstrated its clinical value in terms of reducing the desire to use drugs and the risk of relapse in people who received inpatient or outpatient treatment in an addiction clinical center [20]. However, this type of program requiring an 8-week investment would be difficult to integrate into a prevention approach aimed at regulating alcohol consumption and binge drinking in the student population because of its cost in terms of time and the mobilization of healthcare personnel. To date, very few studies have examined the usefulness of a brief intervention based on MM principles as part of a prevention program for students who are not in the process of treatment. To our knowledge, only one exploratory study has attempted to evaluate the effectiveness of a mindfulness-based brief intervention for students with binge drinking [21]. This first study yielded promising results highlighting the effectiveness of MM in decreasing the frequency of BD use and reported negative consequences related to alcohol consumption as well as in increasing feelings of self-efficacy and mindfulness (i.e., the willingness to pay attention to and accept the experience of the present moment as it is). Nevertheless, these results need to be replicated given the relatively small sample size. In addition, this study has some limitations and questions that the ALCOMEDIIT prevention program will attempt to address. Indeed, the ALCOMEDIIT project is not limited to a 1-month follow-up but also includes an exploratory 6-month follow-up offering the possibility to test the potential beneficial effects of the prevention program over time. Apart from the 1- and 6-month follow-up sessions, information concerning alcohol consumption and daily practice of MM can be collected in an ecological way within a mobile application allowing to prevent possible mnemonic biases often generated by retrospective recall of memories at a distance from the event. The ALCOMEDIIT project also places the control group in a brief intervention condition by having all the participants benefit from a motivational interview, thus making it possible to determine the specific benefits linked to the MM intervention.

### Implementation Intentions and volitional help sheets

Second, the potential benefits expected from this practice of MM on the clinical issue of alcohol consumption in the student population could be optimized by the use of implementation intentions (II). This strategy aimed at limiting unwanted automatic responses through the planification of the suppression, avoidance, or replacement of specific stimulus-response associations. For example, in the ALCOMEDIIT project, the aim is to choose different everyday situations frequently associated with alcohol urge or consumption and to plan to practice mindfulness meditation. By automating the practice of a breathing exercise in a tempting situation, it may reduce the desire to drink and the feeling of craving or reduce the consumption of the substance. Indeed, once people are able to use a MM exercise to contain their urge to consume alcohol, they can plan through implementation intentions to develop their MBM skills [22–24] on the one hand, but on the other hand, they can be trained to identify risky situations, even cravings, in order to plan [25] to regulate their consumption by using MM [26]. The implementation intention technique (II) allow to “go beyond” a mere motivation to act that define what the individual wants to do (e.g., “I want to control my alcohol consumption”), by contextualizing this objective and specifying the action modalities to achieve it. In other words, with this technique, the individual link situational cues (e.g., a place, a moment and/or a mental state) to a goal-directed-response (an emotional, cognitive or behavioral response) to be implemented in response to this stimulus (in our case, a GPA exercise to curb the impulsive drinking reaction). This “situational cues/goal-directed response” link is actualized in the verbal format “if the situation X arises, then I will perform the goal-directed response Z” [27, 28]. This association makes the situational cues more cognitively accessible [29] and automates the response enactment [30]. Thus, the implementation of the objective no longer requires additional elaboration or decision making that could be interfered with by other cues.

In short, the ALCOMEDIIT trial will consist of training people to combine two complementary psychological techniques. First, the capacity for mindfulness will be exercised. Then, persons will form implementation intentions to control their consumption (e.g., [31] for an example targeting BD intentions) by identifying alcohol related situational cues and planning to perform actions related to mindfulness skills. In other words, the objective of ALCOMEDIIT is to give people tools to fight their impulsive tendency to drink (through the development of their mindfulness skills) and to provide them with the psychological equipment to automate the use of these tools (through the adoption of intention implementation techniques).

## Methods/design

### Study objectives

The primary purpose of this work is to evaluate the interest of associating mindfulness meditation practices and implementation of self-regulation strategies to optimize their use, with a motivational interview in a prevention program aiming to prevent binge drinking in the student population. The program will be evaluated by regular self-reported follow-up by the subjects on a non-connected mobile application based on an ecological momentary assessment (EMA [32]) in the medium term (+ 1 month) and in an exploratory way in the long term (+ 6 months).

The secondary objectives are, beyond preventing the practice of BD, to explore the potential further beneficial effects of this prevention program in the medium term (1 month) and long term (6 months; exploratory) on alcohol-related elements (weekly alcohol use, BD, motivation to regulate alcohol use, craving).

### Study design and setting

All the study design and setting (see SPIRIT Checks in Additionnal File 1) has been elaborated by the trial steering committee (TSC) which is made up of the research team who authored this article, with the exception of Messrs Leveneur and Lacherez, who were respectively responsible for the development and design of the ALCOODUC mobile app. The TSC, comprising nine people, is made up of academics specializing in prevention issues and field experts specializing in alcohol-related problems and/or the practice of mindfulness meditation. This steering committee met on a monthly basis during the protocol design phase and validated each stage of the protocol submission process, notably at the level of the national ethics committee (see the “Ethics approval and consent to participate” section for a brief description of the French system).

This is a randomized controlled study in parallel groups. It will compare the monthly BD score between the control group and the experimental group, and the allocation ratio is 1:1.

**Fig. 1:**
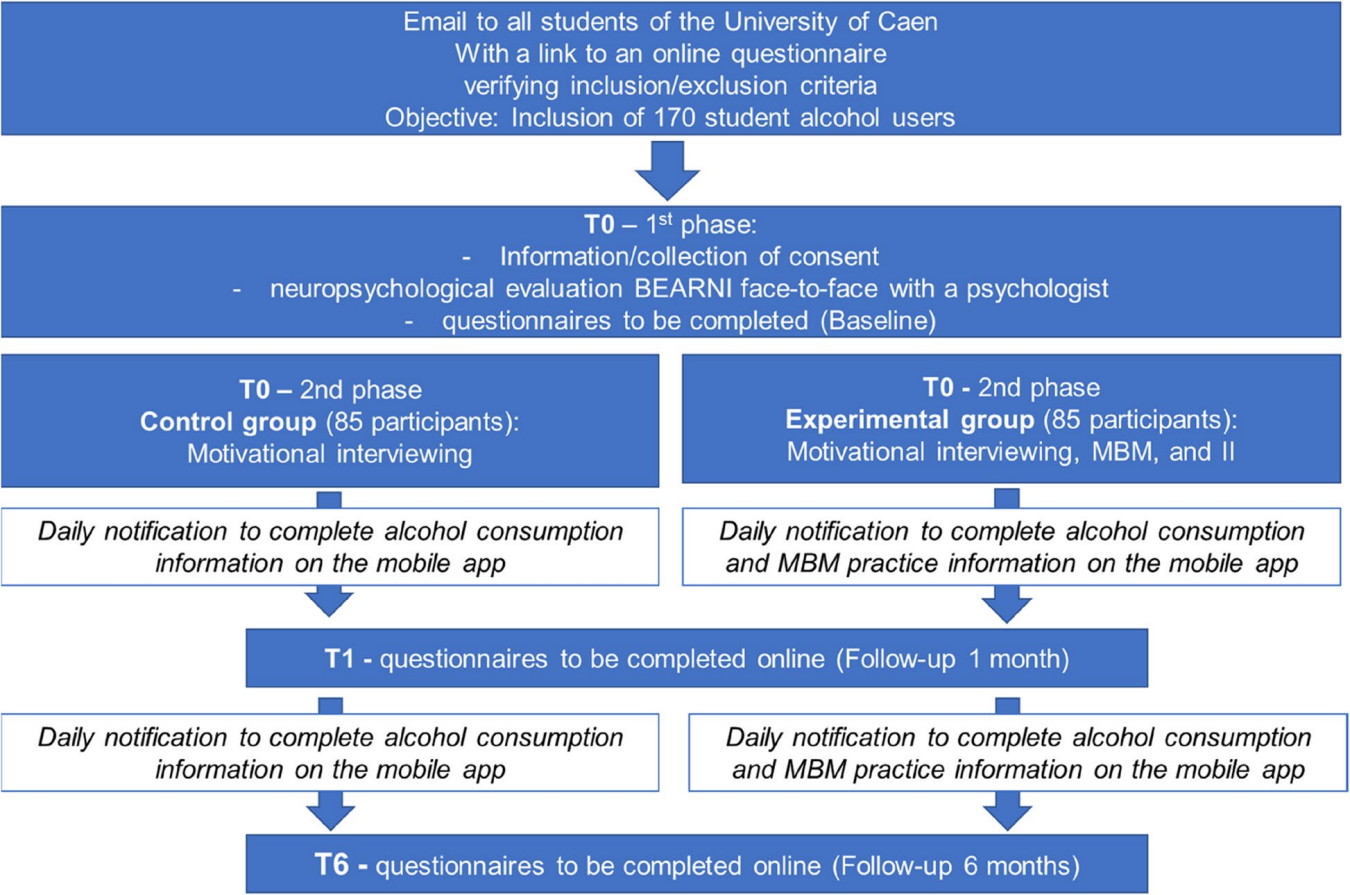
Flux diagram and procedure

One hundred seventy volunteer participants from the University of Caen (France) will be included in this research protocol (see Fig. 1). During the initial visit, after presentation of the study and signature of the consent form presented by one of the two psychologists in charge of the interviews, all participants will complete various online questionnaires (estimated completion time 40 min; LimeSurvey) as well as a screening for neuropsychological impairments using the BEARNI tool (Brief Evaluation of Alcohol-Related Neuropsychological Impairments [33]; estimated completion time of 20 min). Eighty-five participants will benefit from the control condition (i.e., a motivational interview), and 85 other participants will benefit from the experimental condition (i.e., a motivational interview associated with a mindfulness meditation session and intention implementation; estimated duration 1 h).

During follow-ups at 1 month and then at 6 months, alcohol-related questionnaires from the initial visit will be offered again online (LimeSurvey) to the 170 participants to allow for the longitudinal evaluation of these different measures. In addition, all students will be asked to answer regular questionnaires based on Ecological Momentary Assessment (EMA) on an offline mobile application in order to evaluate the monthly BD score (main criterion) and the weekly amount of alcohol consumed and to follow their mindfulness meditation practice (only for the experimental group). This data collection will be done between the different sessions (initial visit “T0” and follow-ups at 1 month “T1” and at 6 months “T6,” the latter being exploratory). All data, including LimeSurvey questionnaires, are hosted on servers at the University of Caen, France.

Randomization will be organized by placing 170 sealed opaques envelopes in which the participant’s group (control vs. experimental) will be specified. After completion of all measures in T0, participants select an envelope from a series of envelopes in a box at their disposal. The ignorance clause will hence be respected. The psychologists in charge of the interviews will be blind until the opening of the envelope drawn by the participant at the end of T0.

The inclusion phase incorporated the possibility of a substantial attrition rate (about 30%) to ensure sufficient statistical power. Therefore, trial exits (refusal to continue^1^, withdrawal of consent, or other) will not be replaced.

### Eligibility criteria

To be eligible, subjects must meet all the defined inclusion criteria. The population, made up of students who consume alcohol recruited within the University of Caen, will be aged between 18 and 30 years, French native speaker, of both sexes, with a binge drinking score^2^ > 1, having signed an informed consent. In addition, subjects meeting any of the following exclusion criteria will not be eligible to participate in the research: pregnant or breastfeeding students, students reporting a history of neurological, neurosurgical, psychiatric or endocrine disease, or infectious disease.

### Recruitment of participants and material

Participation in the study will be proposed to all students of the University of Caen through their institutional email address. In this email, the study will be briefly presented and a link to a LimeSurvey questionnaire will be proposed. On this link, the research and its objectives will be presented in more details, followed by questions on the elements of inclusion and exclusion.

Based on their responses, volunteers may or may not be included in the study sample. An email will inform them of this decision and the volunteers who meet the inclusion criteria will be invited to contact one of the investigating psychologists to schedule the inclusion visit.

### Procedure and measures

**Fig. 2:**
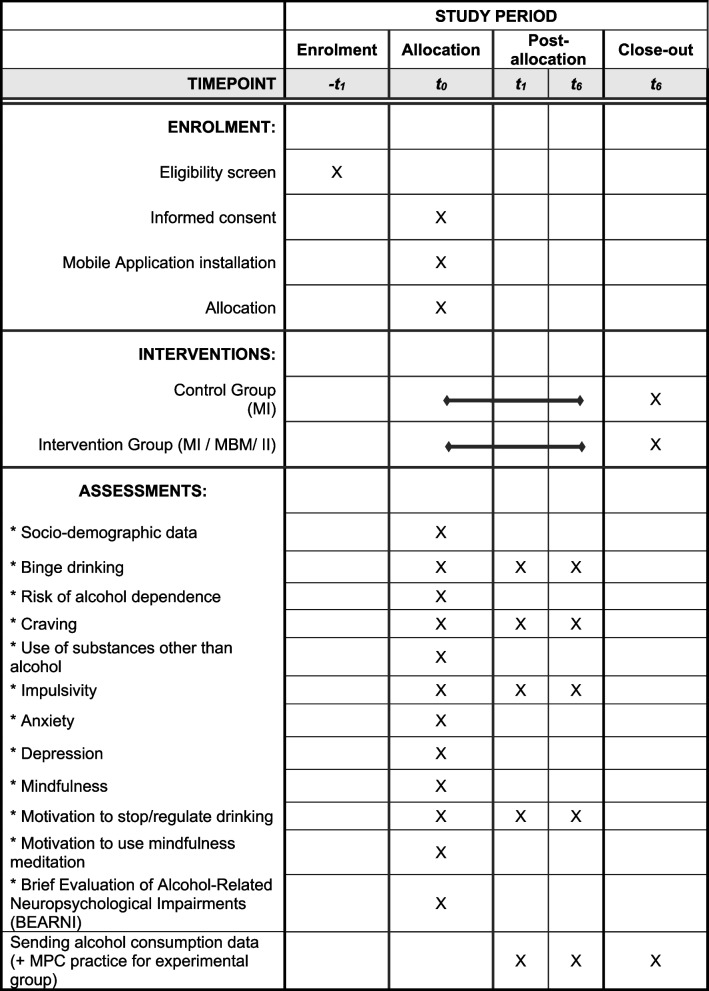
Schedule of enrolment, interventions, and assessments. NB: T1 (+ 1 month). T6 (+ 6 months). MI, motivational interviewing; MBM, mindfulness-based meditation; II, implementation intention

After the online pre-inclusion screening, the inclusion visit will be organized in person (see Fig. 2 for the SPIRIT timeline requirements contents). During this visit, the investigating psychologist will remind the participant of the research and its objectives, will provide the information letter on the foreseeable risks and constraints of the trial, and will collect the participant’s informed consent. An anonymous email address will be created to allow participants to contact their referring psychologist in case of need and to send the data recorded in the mobile application while maintaining their anonymity. This inclusion visit also includes the presentation of the ALCOODUC mobile application allowing all participants to regularly record their alcohol consumption and its modalities during the next 6 months and for the participants of the experimental condition to record their MBM-based practice rhythm as well as the programming of subsequent solicitations. The details of the questions integrated in the mobile application are available on osf: https://osf.io/3n4c6/.

During the first experimental period (T0), both a screening for neuropsychological disorders and an online questionnaire including various psychological measures^3^ will be proposed.

A screening for *neuropsychological disorders* will be offered to all participants using the BEARNI tool (Brief Evaluation of Alcohol-Related Neuropsychological Impairments [33]). BEARNI was specifically designed to screen for cognitive and motor deficits in individuals with alcohol use disorders, including deficits in episodic memory, working memory, executive functions, visuospatial abilities, and ataxia. It contains five subtests: a verbal episodic memory subtest (maximum score: 6 points), an alphabetic span subtest assessing verbal working memory (maximum score: 5 points), an alternating verbal fluency subtest assessing flexibility abilities (maximum score: 6 points), a five complex figures subtest assessing visuospatial abilities (maximum score: 5 points), and an ataxia subtest assessing balance (maximum score: 8 points). The BEARNI tool provides six scores: five sub-scores (one for each of the sub-tests) and a total score (maximum score: 30 points).

#### Socio-demographic data

Information on age, sex, height, weight, and academic year will be collected.

#### Alcohol consumption

Information on age of onset, number of drinks per week (weekly alcohol consumption (WAC)), and type of alcohol will be collected.

#### Binge drinking practices

As indicated earlier, BD score [34] is calculated on the basis of three distinct elements (Q1: “number of average standard drinks per hour (containing about 10g of pure alcohol in France)”; Q2: “number of drunken episodes in the year”; and Q3: “percentage of drunken episodes among the occasions of drinking”). The score results from the following weighting: (4 × Q1) + Q2/2 + (0.2 × Q3). This score considers both the quantity and frequency of drinking, thus integrating the dimension of repeated alcohol withdrawal. To note, the index will need modification to measure BD practice at 1 month and 6 months after the program. Specifically, at T1 (+ 1 month), the BD score will be based on a 1-month-based Q2: “number of episodes of drunkenness in the last month” which will consequently modify the formula (4 × Q1) + Q2 × 6 + (0.2 × Q3). At T6 (+ 6 month), the BD score will be based on a 6-month-based Q2: “number of drunken episodes in the last 6 months” which will consequently modify the formula (4 × Q1) + Q2 + (0.2 × Q3).

#### Risk of alcohol dependence

AUDIT Questionnaire (Alcohol Use Disorders Identification Test) is a 10-question measure designed to identify people who are at risk of developing alcohol-related problems or who are actually experiencing such problems [35]. The AUDIT has been validated and recommended as an effective measure for alcohol among students [36].

#### Craving

One item was included to check the possible craving frequency episodes experienced by the participant.

#### Use of substances other than alcohol

For each other substance (tobacco, cannabis, ecstasy/MDMA, amphetamines, cocaine, heroin, new synthetic drugs excluding ecstasy), participants will be asked to provide a binary response regarding their use (no vs. yes).

#### Impulsivity

Impulsivity is measured using the French version of the short UPPS scale assessing five facets of impulsivity [37]: Negative vs. Positive Urgency (i.e., the tendency to act rashly to regulate a negative vs. positive emotion), Lack of Premeditation (i.e., the tendency to favor immediate reward options without considering the potential consequences of the action), Lack of Perseverance (i.e., poorer concentration on boring or difficult tasks and increased distraction), and Sensation Seeking (i.e., the tendency to seek a new or exciting experience). Participants are asked to respond to 20 statements on a Likert scale from 1 = strongly agree to 4 = strongly disagree.

#### Anxiety

Anxiety is assessed using the French version of the State-Trait Anxiety Inventory (STAI [38]). It consists of 20 items ranging from 1 = no to 4 = yes.

#### Depression

Depressive symptoms are assessed using the French version of the Beck Depression Inventory (BDI-13 [38]). It consists of 13 items (*α* = .87) each composed of four statements reflecting varying degrees of severity of the depression symptom.

#### Mindfulness

The Five Facets Mindfulness Questionnaire (FFMQ [39, 40]) is a 39-item measure of a person’s level of dispositional mindfulness (i.e., personality trait). Participants are asked to respond on a scale ranging from 1 = never or very rarely true to 5 = very often or always true. The FFMQ includes 5 subscales: observation (8 items), description (8 items), acting consciously (8 items), non-judgmental (8 items), non-reactivity (7 items). The sum of the 39 items assesses overall dispositional mindfulness.

#### Motivation to stop/regulate drinking (Readiness to Change-Alcohol, RTC-Alcohol)

In order to evaluate the participants’ readiness to change in their behavior towards alcohol, we will administer the RTC-Alcohol questionnaire to all the students included in the study. This self-assessment based on the transtheoretical model of behavior change [37, 38] is recognized for its reliability and validity. The RTC-Alcohol has three different scales that correspond to an assessment of the three main stages of behavioral change: the pre-contemplation, contemplation, and action stages. Based on the person’s score on each of these scales, the experimenter determines which stage of behavior change the person is in.

#### Motivation to use mindfulness meditation (RTC-Meditation)

In order to assess participants’ behavioral changes regarding the practice of mindfulness meditation, we will offer the RTC-Meditation questionnaire (based on the RTC-Alcohol) to all students included in the study.

Once the neuropsychological evaluation and all the questionnaires have been completed (estimated duration 50 min), on the basis of a random assignment (see randomization procedure with envelopes), the participant will be integrated into the control group or the experimental group. Regardless of the group, all participants will participate to a motivational interviewing session. Only the experimental group will participate in a mindfulness meditation session associated with an implementation intention session.

### Interventions

Distinct interventions are proposed according the allocated group. The framework trial is to test the superiority of the intervention proposed in the experimental group compared to the intervention proposed in the control group.

#### Control group

Participants in the control group will receive no specific psycho-social intervention except that they will benefit from a motivational interview with the psychologist who welcomed them for inclusion.

#### Experimental group

Similar to the control group, participants will benefit from a motivational interview with the psychologist who welcomed them for inclusion. Yet, this motivational interview will be completed by two activities: mindfulness-based initiation and implementation intention exercises.

The mindfulness meditation session is structured in four steps. The session begins with the meditation called “grape exercise” associated with the “sound meditation.” In a second step, the principles of mindfulness meditation (conceptualization of MBM, autopilot and acceptance) are explained to the participants by the psychologist. The session continues with two exercises, the first named “Sitting Meditation Space on thoughts” and the second “SOBER Breathing Space in the face of 1 Challenge situation.” At the end of the session, an explanation on the memo sheet available on the mobile application is given to the participants so that they can continue their practice if a risky substance use situation arises.

The implementation intention session follows the MBM initiation. It focuses on the implementation of two behaviors, on the one hand, regular MBM-based training (II-1) and, on the other hand, the use of MBM in risky drinking situations (II-2). The two systematic phases related to an II process are specific to each of the two implementations (all details available on osf: https://osf.io/3n4c6/.

II-1 deals with the implementation of a daily and/or regular use of MBM. It focuses first on the identification by the participant of a situation, in terms of place, moment of day, and context, which represents an opportunity to reach the goal of practicing MBM on a regular basis, or an obstacle to reaching this goal (II-1a). Then, the participant is invited to identify a thought or behavior that is relevant, for him or her and in this situation that he or she has chosen, to seize this opportunity or overcome this obstacle (II-1b). On this basis, the participant is invited to explicitly formulate the link between these two elements in the linguistic format “If (II-1a), then (II-1b),” to copy it once, to repeat it quietly in his/her head 3 times while trying to represent the scene concretely. The participant must repeat the exercise a second time by proposing another “II-1a” and another “II-1b.”

The II-2 on the implementation of the use of MBM in risky situations is structured in the same way. First, the participant is asked to identify a situation, in terms of location, time of day, or context, that represents an opportunity to achieve the goal of regulating one’s drinking with the help of the MBM or an obstacle to achieving this goal (II-2a). The participant is then asked to identify a thought or behavior that is relevant to him or her in the situation he or she has chosen, in order to seize this opportunity or overcome this obstacle (II-2a). On this basis, the participant is invited to explicitly formulate the link between these two elements in the linguistic format “If (II-1a), then (II-1b),” to copy it once, to repeat it quietly in his/her head 3 times while trying to represent the scene concretely. The participant must repeat the exercise a second time by proposing another “II-2a” and another “II-2b.”

### Outcomes

Primary and secondary outcomes will be assessed three times (see Fig. 2 for an overview of outcomes and data time points). Unless they request it, participants will not be physically seen by the psychologists during follow-ups. They will be asked by email 1 month (T1) and 6 months (T6) after the inclusion visit to answer the online questionnaires again and, if they accept, to validate the sending of their alcohol consumption data and, for the participants of the experimental condition, the sending of their MBM data through the mobile application. The participant code included in the file will allow the scientific team to associate the new data with the previous data of this specific participant.

### Statistical considerations

#### Power analysis

At an alpha risk of .05, the number of 120 subjects in total is sufficient to obtain a power greater than .80. This estimate is derived from a 3000 iteration Monte Carlo simulation based on a baseline of real data collected in November 2021 (*N* = 1917) with a Gamma distribution. In this simulation are parameterized an average decrease of 10% of the BD score in control group and an average decrease of 20% for the experimental group with an intra-subject variability (SD) linearly depending on the individual baseline (estimated to be ¼ of the baseline value + 0.5).

Considering a potential attrition rate of approximately 30%, 170 subjects will be included in the study at T0.

#### Data management and analysis plan

Missing data will exclude the corresponding participant from the analysis.

All models will be tested using R software and the expected level of statistical significance is .05. The statistical analyses will be conducted in two main stages and an additional exploratory stage. All details of the Statistical Analysis Plan (SAP) are included in a supplementary file available online (https://osf.io/3n4c6/files/osfstorage ).

A first step will consist in checking that the experimental and control groups are matched at T0. We will use *χ*^2^ tests of independence and Student *t*-tests to check that the randomization did result in two groups that were initially matched in terms of age, sex, AUDIT score, trait anxiety level, and depression level. Should this not be the case, the culprit variables will be controlled as additional factors/covariates in all subsequent analyses (although sex and AUDIT score will be part of the model in any case).

A second step will consist in conducting the main analyses. Recall that by “five dependent variables (DVs),” we mean here binge drinking score (BD), weekly alcohol consumption (WAC), craving, impulsivity, and readiness to change (RC). Prior to the main analyses, we will check whether there is a significant relationship between age and the baseline scores of any of the five DVs. We expect the age range to be rather small apart from potential serious outliers, in a sample that is not particularly large, and we do not expect age to play a noticeable role on most of the DVs, except for BD scores where it is suspected that binge drinking behaviors are most common in the younger population. For these reasons, we want to avoid adding age as a covariate unless the data show that we really have to. We will conduct five similar general linear analyses, with the following variables: dependent variable: evolution scores (T0 → T1) of one of the five DVs; factors: condition, sex, condition*sex; covariates: AUDIT score, baseline of the DV at hand. Covariate(s) that will be added if either they are significantly related to the baseline scores of the DV at hand, or the two groups were not matched at T0 with respect to them: age, trait anxiety level, depression level. We add as a covariate the baseline of the DV at hand because we expect that the higher level of self-consciousness brought by the very fact of taking part in this demanding study might trigger an effect of regression to the mean, by which people with a larger baseline score (and hence a larger margin for improvement) would show better results no matter in what condition they were placed.

In the main analysis, the use of evolution scores as a DV has a number of consequences. First, the effect of condition as reported in the analysis can actually be interpreted as an interaction effect between condition and the within-subject time factor. Second, the condition factor will be dummy-coded (experimental = 1; control = 0) so that the intercept of the model corresponds to the average evolution in the control group, in other words the effect of motivational interviewing alone (plus the fact of having to report your data on a daily basis), while the condition effect will correspond to the added value of the mindfulness training. We will investigate the simple effects of condition and sex, should their interaction turn out to be significant. All these analyses will be repeated using the evolution scores T0 → T6 as well.

### Dissemination policy

The results of the study will be disseminated by the research team involved at various levels. On the one hand, local alcohol prevention organizations (e.g., university prevention services) will be informed of the results in order to jointly examine the transferability of the ALCOMEDIIT program to their own prevention interventions. On the other hand, the scientific community as well as funders will be informed of the results through communications in conferences dedicated to public health and alcohol issues and through scientific publications in peer-reviewed journals.

## Discussion

ALCOMEDIIT protocol aims to assess an innovative program for the prevention of BD practices based on motivational interviewing (MI) associated with implementation intentions (II) and mindfulness meditation (MBM) in a student environment.

The study presents several strengths. First, the alarming data on BD practice, even with a promising decrease in alcohol consumption over the last decade in students’ environment, makes it necessary to develop and test new optimized prevention practices. While environmental levers are necessary and the responsibility of public policy, current scientific knowledge about the psychological processes underlying BD allows for *theory-based prevention programs* (e.g., 39) to optimize the potential effects of more conventional programs. In this perspective, ALCOMEDIIT protocol aims to assess an innovative program for the prevention of BD practices. For the first time, to our knowledge, a conventional approach relying on motivational interviewing is associated with a double innovation: a brief introduction to mindfulness is associated with implementation intentions in order to optimize its use. If our theoretical reasoning is empirically validated in this randomized controlled trial, participants will be able to improve their impulsivity control and thereby better regulate their drinking by reducing BD use. Second, the research *team* is composed of specialists in addiction (researchers and physician), in mindfulness (researcher and psychologist), in behavior change theories and techniques (researcher). This theoretical, methodological, and experiential diversity is further supported by a collection of complementary data (e.g., 12) that allows us to precisely target the relevant determinants of BD behavior specific to the student population. Third, the development with an informatician and a professional graphist of a *specific mobile application* dedicated to the data collection allowed us to have a tool perfectly adapted to the ALCOMEDIIT protocol. Indeed, if the mobile application sends a daily notification to the participants to propose them to indicate their consumption, it also facilitates the practice of meditation for the participants of the experimental group by proposing them, integrated in the interface, the MBM exercises to which they were initiated (see screenshots on the osf material sharing platform: https://osf.io/3n4c6/).

Yet, some limitations have to be acknowledged. First, only recruiting volunteers may create a sampling bias. However, this procedure is ideal for the use of implementation intentions. Many researches showed that being motivated by the goal-directed behavior is a moderator, and even a pre-requisite, of implementation intentions effects: this strategy facilitate goal achievement only when people are motivated to act [41, 42]. This condition would here be satisfied since voluntarily engaging in the present study should imply to be motivated by the target behavior, meaning reducing alcohol consumption and use of binge drinking. Lastly, we cannot exclude different *attrition rates* between the intervention and control groups. This difference could lead to biased estimates of the intervention’s effects. If data would evidence such a difference, appropriate econometric techniques will be mobilized to correct for attrition bias [43]. In terms of *potential risks*, motivational interviewing, which aims to reduce resistance to change by exploring the ambivalence of the person with regard to his or her substance use behavior, is used within the framework of the classic treatment of addiction and does not involve any particular risks. As far as mindfulness meditation is concerned, too intensive or inappropriate practice can exceptionally lead to an excessive regulation of emotions and thus to an alteration of well-being, especially in people suffering from psychological disorders. To counter this potential risk, within the framework of the ALCOMEDIIT protocol, people suffering from psychiatric disorders will be excluded and the practice of MBM will be carried out under the supervision of a psychologist specifically trained in MBM.

The results of the program will be of significant interest both from an applied and a scientific point of view. On the one hand, it should help to implement public health interventions that are effective at helping students to regulated their impulsivity and consequently to regulate their BD practices specifically driven by impulsivity. On the other hand, this is the first association between motivational interviewing and two antecedent-focused strategies that aim to avoid exposure to or activation of automatic (i.e., impulsive) responses that lead to alcohol consumption, namely intention implementation and mindfulness-based meditation.

### Trial status

The study started recruiting in October 3, 2022, and is actually under progress (*N* = 98/170). The study completion will be July 4, 2024. The protocol number is 2.0 (ID-RCB 2022-A00983-40) and has been validated on June 23, 2022.

### Supplementary Information

**Additional file 1.** SPIRIT 2013/2022 Checklist: Recommended items to address in a clinical trial protocol and related documents

## Data Availability

As it is a study protocol publication, data are not yet available. Nevertheless, once collected, the datasets generated and/or analyzed during the current study are available in the open science framework on: https://osf.io/3n4c6/.

## Abbreviations

BD: Binge drinking
MM: Mindfulness meditation
MI: Motivational interview
II: Intention implementation

## Footnotes

1 Participants have the right to refuse to participate in the study or to withdraw their consent at any time without incurring any liability. This information is made clear verbally at the first encounter and also on the consent form.

2 The BD score is calculated on the basis of 3 separate items (Q1: “average number of drinks per hour”; Q2: “number of binge drinks in the past month”; and Q3; “percentage of binge drinks among the occasions of drinking in the past month”). The score results from the following weighting: (4 × Q1) + Q2 + (0.2 × Q3). This score considers both the quantity and frequency of consumption, thus integrating the dimension of repeated alcohol withdrawal.

3 Details of the items in each questionnaire are presented on osf: https://osf.io/3n4c6/
