# Supplementary material for: A prevention program for binge drinking among students based on mindfulness and implementation intention (ALCOMEDIIT): a randomized controlled trial
Source: Trials. 2024 Jan 2;25:1. doi: 10.1186/s13063-023-07887-9 (PMC10759352; doi:10.1186/s13063-023-07887-9)
Supplement: Supplementary file 1 — Additional file 1. SPIRIT 2013/2022 Checklist: Recommended items to address in a clinical trial protocol and related documents [file 13063_2023_7887_MOESM1_ESM.docx]

**Additional file 1.** SPIRIT 2013/2022 Checklist: Recommended items to address in a clinical trial protocol and related documents*


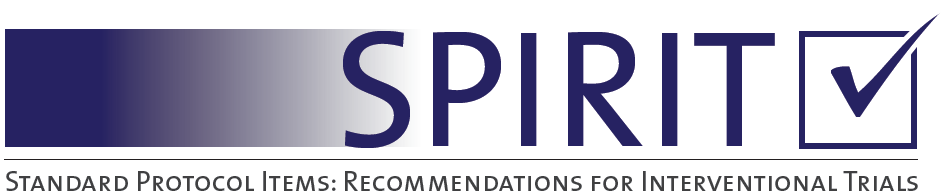


| **Section/item** | **ItemNo** | **Description SPIRIT 2013 Item** | **Description SPIRIT 2022 Item** | **Addressed in section** |
| --- | --- | --- | --- | --- |
| **Administrative information** | | |  |  |
| Title | 1 | Descriptive title identifying the study design, population, interventions, and, if applicable, trial acronym |  | Title page (p.1) |
| Trial registration | 2a | Trial identifier and registry name. If not yet registered, name of intended registry |  | Abstract (p. 2) |
|  | 2b | All items from the World Health Organization Trial Registration Data Set |  | See ClinicalTrials.gov (p. 2) |
| Protocol version | 3 | Date and version identifier |  | Abstract (p. 2) and Trial Status (p. 14) |
| Funding | 4 | Sources and types of financial, material, and other support |  | Funding (p. 15) |
| Roles and responsibilities | 5a | Names, affiliations, and roles of protocol contributors |  | Authors’ contributions (p. 15) |
|  | 5b | Name and contact information for the trial sponsor |  | Ethics approval (p. 14)  In France, the sponsor is called “Promoteur de l’étude” and constitutes the guarantee for ethical issues. |
|  | 5c | Role of study sponsor and funders, if any, in study design; collection, management, analysis, and interpretation of data; writing of the report; and the decision to submit the report for publication, including whether they will have ultimate authority over any of these activities |  | Funding (p. 15) |
|  | 5d | Composition, roles, and responsibilities of the coordinating centre, steering committee, endpoint adjudication committee, data management team, and other individuals or groups overseeing the trial, if applicable (see Item 21a for data monitoring committee) |  | Study design and Setting (p. 5) |
| **Introduction** |  |  |  |  |
| Background and rationale | 6a | Description of research question and justification for undertaking the trial, including summary of relevant studies (published and unpublished) examining benefits and harms for each intervention |  | Background (pp. 3/4) |
|  | 6b | Explanation for choice of comparators |  | Background (p. 3) |
| Objectives | 7 | Specific objectives or hypotheses |  | Background (p. 5) |
| Trial design | 8 | Description of trial design including type of trial (eg, parallel group, crossover, factorial, single group), allocation ratio, and framework (eg, superiority, equivalence, noninferiority, exploratory) |  | Study design and setting (p. 5)  Interventions (p. 10) |
| **Methods: Participants, interventions, and outcomes** | | |  |  |
| Study setting | 9 | Description of study settings (eg, community clinic, academic hospital) and list of countries where data will be collected. Reference to where list of study sites can be obtained |  | Study design and setting (pp. 5/6) |
| Eligibility criteria | 10 | Inclusion and exclusion criteria for participants. If applicable, eligibility criteria for study centres and individuals who will perform the interventions (eg, surgeons, psychotherapists) |  | Eligibility criteria (p. 7) |
| Interventions | 11a | Interventions for each group with sufficient detail to allow replication, including how and when they will be administered |  | Interventions (pp. 10/11) |
|  | 11b | Criteria for discontinuing or modifying allocated interventions for a given trial participant (eg, drug dose change in response to harms, participant request, or improving/worsening disease) |  | N/A  There is no justification for interrupting or modifying the interventions allocated to a given trial participant, and therefore no established criteria on this point. |
|  | 11c | Strategies to improve adherence to intervention protocols, and any procedures for monitoring adherence (eg, drug tablet return, laboratory tests) |  | The strategy for improving adherence to the intervention protocol is to provide a mobile application including tools for practicing mindfulness, and the procedures for monitoring adherence are identifiable by the use of a daily ecological momentary assessment on this practice for participants in the control group (p. 7). |
|  | 11d | Relevant concomitant care and interventions that are permitted or prohibited during the trial |  | Participation in other studies involving mindfulness meditation during the ALCOMEDIIT trial is not permitted (see consent form on osf <https://osf.io/3n4c6/> as indicated p. 7). |
| Outcomes | 12 | Primary, secondary, and other outcomes, including the specific measurement variable (eg, systolic blood pressure), analysis metric (eg, change from baseline, final value, time to event), method of aggregation (eg, median, proportion), and time point for each outcome. Explanation of the clinical relevance of chosen efficacy and harm outcomes is strongly recommended |  | Outcomes (p. 11) |
|  | 12.1 |  | Provide a rationale for the selection of the domain for the trial’s primary outcome | Procedure and measures (p. 9) |
|  | 12.2 |  | If the analysis metric for the primary outcome represents within-participant change, define and justify the minimal important change in individuals | Statistical considerations (p. 11) |
|  | 12.3 |  | If the outcome data collected are continuous but will be analyzed as categorical (method of aggregation),  specify the cutoff values to be used | The data are continuous and analyzer as continuous. |
|  | 12.4 |  | If outcome assessments will be performed at several time points after randomization, state the time points that will be used for analysis | The main criteria is the BD score after 1 month. In complement, the BD score will also exploratorily be evaluated 6 months after the intervention (p. 9) |
|  | 12.5 |  | If a composite outcome is used,  define all individual components  of the composite outcome | Procedures and measures (p. 9) |
| Participant timeline | 13 | Time schedule of enrolment, interventions (including any run-ins and washouts), assessments, and visits for participants. A schematic diagram is highly recommended (see Figure) |  | Figure 2 (p. 7) |
| Sample size | 14 | Estimated number of participants needed to achieve study objectives and how it was determined, including clinical and statistical assumptions supporting any sample size calculations |  | Power analysis (p. 11) |
|  | 14.1 |  | Define and justify the target  difference between treatment groups  (eg, the minimal important difference) | Power analysis (p. 11) |
| Recruitment | 15 | Strategies for achieving adequate participant enrolment to reach target sample size |  | Recruitment of participants and material (pp. 6/7) |
| **Methods: Assignment of interventions (for controlled trials)** | | |  |  |
| Allocation: |  |  |  |  |
| Sequence generation | 16a | Method of generating the allocation sequence (eg, computer-generated random numbers), and list of any factors for stratification. To reduce predictability of a random sequence, details of any planned restriction (eg, blocking) should be provided in a separate document that is unavailable to those who enrol participants or assign interventions |  | N/A |
| Allocation concealment mechanism | 16b | Mechanism of implementing the allocation sequence (eg, central telephone; sequentially numbered, opaque, sealed envelopes), describing any steps to conceal the sequence until interventions are assigned |  | Study Design and setting (random drawing of opaque, sealed envelopes; p. 6) |
| Implementation | 16c | Who will generate the allocation sequence, who will enrol participants, and who will assign participants to interventions |  | recruitment of participants and material (pp. 6/7) |
| Blinding (masking) | 17a | Who will be blinded after assignment to interventions (eg, trial participants, care providers, outcome assessors, data analysts), and how |  | The intervention is and explicit psycho-social intervention and thus after random allocation either to control or to the experimental condition, both participants and experimenters know the participant’s allocated intervention.  study design and setting (p. 6) |
|  | 17b | If blinded, circumstances under which unblinding is permissible, and procedure for revealing a participant’s allocated intervention during the trial |  | N/A (cf. answer above) |
| **Methods: Data collection, management, and analysis** | | |  |  |
| Data collection methods | 18a | Plans for assessment and collection of outcome, baseline, and other trial data, including any related processes to promote data quality (eg, duplicate measurements, training of assessors) and a description of study instruments (eg, questionnaires, laboratory tests) along with their reliability and validity, if known. Reference to where data collection forms can be found, if not in the protocol |  | Procedure and measures (pp. 7/8) Outcomes (p. 11) |
|  | 18a1 |  | Describe what is known about the  responsiveness of the study  instruments in a population similar to the study sample | Background (pp. 3/4) |
|  | 18a2 |  | Describe who will assess the  outcome (eg, nurse, parent) | Participant will assess themselves the outcome with the mobile phone application including the daily ecological momentary assessment  Procedure and measures (p. 7) |
|  | 18b | Plans to promote participant retention and complete follow-up, including list of any outcome data to be collected for participants who discontinue or deviate from intervention protocols |  | Procedure and measures (pp. 7/8) Outcomes (p. 11) |
| Data management | 19 | Plans for data entry, coding, security, and storage, including any related processes to promote data quality (eg, double data entry; range checks for data values). Reference to where details of data management procedures can be found, if not in the protocol |  | Data management (pp. 11/12) |
| Statistical methods | 20a | Statistical methods for analysing primary and secondary outcomes. Reference to where other details of the statistical analysis plan can be found, if not in the protocol |  | Analysis plan (pp. 11/12) |
|  | 20a.1 |  | Describe any planned methods to account for multiplicity in the analysis  or interpretation of the primary and secondary outcomes (eg, coprimary  outcomes, same outcome assessed at multiple time points, or subgroup analyses of an outcome) | Analysis plan (pp. 11/12) |
|  | 20b | Methods for any additional analyses (eg, subgroup and adjusted analyses) |  | Analysis plan (pp. 11/12) |
|  | 20c | Definition of analysis population relating to protocol non-adherence (eg, as randomised analysis), and any statistical methods to handle missing data (eg, multiple imputation) |  | Analysis plan (pp. 11/12) |
| **Methods: Monitoring** | | |  |  |
| Data monitoring | 21a | Composition of data monitoring committee (DMC); summary of its role and reporting structure; statement of whether it is independent from the sponsor and competing interests; and reference to where further details about its charter can be found, if not in the protocol. Alternatively, an explanation of why a DMC is not needed |  | N/A  No Data Monitoring Committee (DMC) is involved. Data comes from participants' own mobile applications. |
|  | 21b | Description of any interim analyses and stopping guidelines, including who will have access to these interim results and make the final decision to terminate the trial |  | N/A  The sponsor requires that no interim analyses be conducted. The end of recruitment can take place when we have reached the statistical power required to evaluate the effect of the intervention at 1 month. |
| Harms | 22 | Plans for collecting, assessing, reporting, and managing solicited and spontaneously reported adverse events and other unintended effects of trial interventions or trial conduct |  | N/A  Given the type of intervention used (psychosocial intervention), no adverse events are envisaged. |
| Auditing | 23 | Frequency and procedures for auditing trial conduct, if any, and whether the process will be independent from investigators and the sponsor |  | N/A  Once again, the data comes from the participants' mobile application, and the participants themselves decide at 1 and 6 months whether or not to share the data with the research team. |
| **Ethics and dissemination** | | |  |  |
| Research ethics approval | 24 | Plans for seeking research ethics committee/institutional review board (REC/IRB) approval |  | Ethics approval (p. 14) |
| Protocol amendments | 25 | Plans for communicating important protocol modifications (eg, changes to eligibility criteria, outcomes, analyses) to relevant parties (eg, investigators, REC/IRBs, trial participants, trial registries, journals, regulators) |  | Ethics approval (p. 14) |
| Consent or assent | 26a | Who will obtain informed consent or assent from potential trial participants or authorised surrogates, and how (see Item 32) |  | Study design and setting (p.5) |
|  | 26b | Additional consent provisions for collection and use of participant data and biological specimens in ancillary studies, if applicable |  | N/A  No collection or use of participant data or biological samples for ancillary studies |
| Confidentiality | 27 | How personal information about potential and enrolled participants will be collected, shared, and maintained in order to protect confidentiality before, during, and after the trial |  | Study design and setting (p. 6) |
| Declaration of interests | 28 | Financial and other competing interests for principal investigators for the overall trial and each study site |  | Competing interests (p. 15) |
| Access to data | 29 | Statement of who will have access to the final trial dataset, and disclosure of contractual agreements that limit such access for investigators |  | Procedure and measures (pp. 7/8) |
| Ancillary and post-trial care | 30 | Provisions, if any, for ancillary and post-trial care, and for compensation to those who suffer harm from trial participation |  | N/A  No ancillary care is envisaged, given the type of intervention (psycho-social) |
| Dissemination policy | 31a | Plans for investigators and sponsor to communicate trial results to participants, healthcare professionals, the public, and other relevant groups (eg, via publication, reporting in results databases, or other data sharing arrangements), including any publication restrictions |  | Dissemination policy (p. 12) |
|  | 31b | Authorship eligibility guidelines and any intended use of professional writers |  | Authors' contributions (p. 15) |
|  | 31c | Plans, if any, for granting public access to the full protocol, participant-level dataset, and statistical code |  | Procedure and measures (p. 7) |
| **Appendices** |  |  |  |  |
| Informed consent materials | 32 | Model consent form and other related documentation given to participants and authorised surrogates |  | The model consent form is available on request and all other documents are available on osf (p. 7) |
| Biological specimens | 33 | Plans for collection, laboratory evaluation, and storage of biological specimens for genetic or molecular analysis in the current trial and for future use in ancillary studies, if applicable |  | N/A  No biological specimens are included in the study. |

*It is strongly recommended that this checklist be read in conjunction with the SPIRIT 2013 Explanation & Elaboration for important clarification on the items. Amendments to the protocol should be tracked and dated. The SPIRIT checklist is copyrighted by the SPIRIT Group under the Creative Commons “[Attribution-NonCommercial-NoDerivs 3.0 Unported](http://www.creativecommons.org/licenses/by-nc-nd/3.0/)” license.

N/A: not applicable.
